# Therapeutic efficacy of an injectable formulation of purinostat mesylate in SU-DHL-6 tumour model

**DOI:** 10.1080/07853890.2022.2045347

**Published:** 2022-03-04

**Authors:** Zejiang Zhu, Jiaolin Wen, Yaohui Xu, Heying Pei, Dan Li, Minghai Tang, Peng Bai, Jun He, Zhuang Yang, Lijuan Chen

**Affiliations:** a State Key Laboratory of Biotherapy and Cancer Center, National Clinical Research Center for Geriatrics, West China Hospital of Sichuan University, Chengdu, China; b West China-California Research Center for Predictive Intervention Medicine, West China Hospital, Sichuan University, Chengdu, China; c Chengdu Zenitar Biomedical Technology Co., Ltd, Biomedical innovation Incubation Park, Chengdu, China

**Keywords:** Purinostat mesylate, injectable formulation, SU-DHL-6 cell line, anti-tumour

## Abstract

**Background:** Previous studies have proven that Purinostat Mesylate (PM) is a new HDAC inhibitor and exhibits significant antitumor efficacy. However, the clinical application of PM was greatly limited by its poor solubility in water and low bioavailability.

**Objective:**To increase the solubility of PM through pharmaceutical research, and prepare it into an injection that meets the needs of intravenous use to promote its clinical application.

**Methods**The prepared PM/HP-β-CD inclusion complex was studied by computer simulation, fourier transform infrared spectroscopy (FT-IR), nuclear magnetic resonance (1H-NMR spectroscopy), and scanning electron microscopy (SEM). Then, the antitumor effects of PM/HP-β-CD inclusion complex were studied by in vitro cytotoxicity assay, apoptosis assay, pharmacokinetic study and in vivo antitumor assay.

**Results:**Phase Solubility Analysis revealed that PM and HP-β-CD were compatible and the solubility of PM increased almost 220 times, to 2.02 mg/mL. The interaction mechanism studies revealed that PM could be embedded into the cavity of HP-β-CD through the side of the aminobenzene ring. Cell viability and apoptosis assays showed that PM/HP-β-CD complex maintained the good anti-cancer activity of PM, and PM/HP-β-CD complex has a better anti-tumor effect and lower toxicity than LBH589 and Hyper-CVAD/RTX in vivo. All the results suggest that HP-β-CD can solve the problem of PM administration and provide a way for clinical application of PM.

**Conclusions:** In this study, an injectable formulation of PM in HP-β-CD (10% w/v) was prepared to improve its water solubility. Our research provides a way for clinical administration of PM, which has been under phase I clinical trial for the treatment of relapsed or refractory B-cell-related hematologic malignancies in China and the USA.

**KEY MESSAGES**
- We developed a preparation of Purinostat Mesylate that can be administered intravenously, reducing the toxicity associated with oral administration.
- This preparation has an outstanding therapeutic effect on SU-DHL-6 xenograft tumour, indicating its clinical value, which has been under phase I clinical trial for the treatment of relapsed or refractory B-cell-related haematologic malignancies in China and the USA.

## 1. Introduction

Pathogenic factors of haematological tumours are systemic, diffuse, and unresectable and are more complicated than those of solid tumours [1]. In recent years, it has been found that in developed countries, the incidence of haematological tumours even exceeds that of liver cancer [2] and gastric cancer [3–5]. The overall proportion of B cell-associated tumours in haematological tumours is as high as 80% [6,7], and the subtypes of haematological tumours are derived at different stages of B cell development, and are often associated with gene translocation, gene mutations, and epigenetic abnormalities [4]. Although marketed drugs and mature treatment options are available for B-cell-associated lymphoma, they are still ineffective or have low efficacy for B-cell-associated lymphoma represented by double-hit lymphoma and Burkitt's lymphoma [8]. The current median survival of double-hit lymphoma is less than 12 months [8]. Relevant clinical research has shown that patients with identified double-hit lymphoma treated with a variety of regimens generally have a low survival rate, with an overall median survival rate of only 0.2 to 1.5 years, due to the physical limitations of older patients. Therefore, the importance of developing new drugs is self-evident [9].

The inhibitors of histone deacetylase (HDAC) catalyse the deacetylation of histones and some non-histone lysine residues and play a vital role in the epigenetic and post-transcriptional modification in cells [10]. The high expression or abnormal activity of HDAC enzyme in tumours always leads to epigenetic uncontrolled dysregulation of important functional proteins in the cytoplasm which can be related to cell cycle, apoptosis, autophagy, migration, intracellular substance transport, and metabolism [11]. HDAC enzyme also produces changes in the immune system at the animal level. At present, five HDAC inhibitors have been clinically approved, and the indications are blood system tumours.

Purinostat mesylate (PM), a novel class I and class IIb HDAC inhibitor using the morpholinopurine as the capping group has been reported by our group [12]. PM has a good inhibitory effect on a variety of B cell-associated haematological tumour cells [13], but its clinical applications is greatly limited by poor solubility. Cyclodextrin and its derivatives are often used to improve the solubility of hydrophobic drugs [14]. During the derivatives, 2-hydroxypropyl-β-cyclodextrin (HP-β-CD) is extensively used, which is composed of a hydrophilic surface and a hydrophobic core [15,16]. Such internal hydrophobic cavities are commonly used to increase the solubility, stability, release, and bioavailability of the active drug [12,17]. Besides, HP-β-CD is a safe, nontoxic pharmaceutical excipient approved by the European Medicines Agency and the US FDA [14] for oral, intravenous, topical application, eye administration, and intestinal administration. [18].

In our study, we report a complex of PM and HP-β-CD (PM/HP-β-CD) to generate lyophilised powder soluble in normal saline to improve the solubility, while maintaining the anti-tumour activity of PM as an HDAC inhibitor. The prepared PM/HP-β-CD is characterized by the following methods, including Fourier transform infra-red spectroscopy (FT-IR), nuclear magnetic resonance (^1^H-NMR spectroscopy), and scanning electron microscopy (SEM). Then, we demonstrate that the PM/HP-β-CD has the same antitumour effect as free PM by *in vitro* cytotoxicity assay, apoptosis assay. Besides, the PM/HP-β-CD complex shows certain safety. The anti-tumour results of oral and intravenous administration indicate that PM/HP-β-CD is a suitable formulation for injection. A complete tumour pathologic analysis is also performed.

## 2. Materials and methods

### 2.1. Materials, cell lines, and animals

PM was synthesized as in previous studies in our laboratory. HP-β-CD was purchased from Libang Pharmaceutical (Xi’an, China). Dimethyl sulfoxide (DMSO) and MTT were purchased from Sigma-Aldrich Inc. Acetonitrile (HPLC grade) was from Fisher Chemical (Loughborough, UK). Water was deionised by the Mili-Q plus system (Millipore). All the other reagents and solvents involved are no less than analytic reagent (AR) grade.

The SU-DHL-6 cells were from the American Type Culture Collection (ATCC. Rockville, MD) and cultured in RPMI 1640 medium with 10% FBS (Gibco, Thermo Fisher Scientific) and 1% antibiotics (Gibco, Thermo Fisher Scientific) at 37 °C in a humidified incubator with 5% CO2.

The female NOD/SCID mice and the SD rats were purchased from the HFK Bio-Technology, Co., LTD (Beijing, China). The animals were separately housed in the standard feeding environment. All the animal experiments were followed protocols approved by the Institutional Animal Care and Treatment Committee of Sichuan University (Chengdu, China), and also followed the ARRIVE guidelines.

### 2.2. Quantification of purinostat mesylate

The concentration and purity of PM were detected by reverse-phase high-performance liquid chromatography (HPLC, Waters 2695 and Waters 2998) as the previous method [13]. A chromatographic column (C18, Waters, 4.6 mm × 150 mm) was used. The gradient detection method of HPLC is shown in Table 1, mobile phase A is acetonitrile, and mobile phase B is a water solution containing 0.5% acetic acid and 10 mM ammonium acetate.

**Table 1.** The gradient detection method of HPLC.

Time (minutes)	Mobile phase A (%)	Mobile phase B (%)
0	72	28
15	72	28
25	45	55
30	45	55
31	72	28
40	72	28

### 2.3. Phase solubility analysis

Excess PM were added into 6 mL HP-β-CD solutions ranged from 0 to 150 mg/mL [14,19]. The mixture solution was stirred at 1000 rpm for 24 h at 37 °C and filtered using 0.22 μm Millipore filtration. The apparent solubility of PM was calculated, and the stability constant (Ks) was calculated as the Higuchi-Connors equation, Equation (a) [20]. The Gibbs free energy (ΔG) was calculated as Equation (b) [21], R represents the general gas constant and T represents the Kelvin temperature.

(a) Ks=slope/(S0×(1–slope))

(b) ΔG=−RTlnKs

### 2.4. Computer simulation

The chemical molecular structure was drawn by ChemDraw software and saved in SDF format. Then, the Discovery Studio software package was used to hydrotreat the drug molecules, and the CHARMm force field was used for energy optimisation. The steepest descent method with the maximum step of 5000 steps was used. The average gradient tolerance is 0.01 kcal/(mol*Angstrom). The drug molecule is docked into the HP-β-CD molecule using the CDOCKER module of Discovery Studio software, and the optimal conformation of the docking complex is preserved.

The preserved complex structure was imported into the Schrodinger software, and the molecular dynamics simulation was performed using the Desmond module. The processes are as follows: the OPLS3 force field parameter of Shrodinger was used to minimise the complexity of the drug molecule and HP-β-CD. A 10 Å explicit TIP3P cube water box was built along the periphery of the solute, and Na + was added as a counter ion to maintain the total electric charge of the system at 0. Meanwhile, 0.15 M NaCl was added to simulate a humoral environment. The traditional molecular dynamics simulation was calculated by the Desmond program.

### 2.5. Preparation of PM/HP-β-CD complex

PM/HP-β-CD complex was prepared with a simple dissolution method. Firstly, 10% HP-β-CD was added into deionised water and stirred for 2 h at 37 °C. Secondly, PM was added and stirred for 24 h. After filtration through a 0.22-μm filtration membrane to remove the excess PM, the filtered solution was mixed with lyophilised excipients including arginine, meglumine, and mannitol, freeze-dried in a penicillin bottle and stored at 2 ∼ 8 °C. The inclusion complex can be re-dissolved in normal saline injection to obtain a clear yellow solution. Besides, PM and HP-β-CD were physically mixed with the molar ratio at 1:1 as the control group.

### 2.6. Characterization of PM/HP-β-CD complex

#### 2.6.1. FTIR

The infra-red spectra of PM, HP-β-CD, PM/HP-β-CD inclusion complex, and PM/HP-β-CD physical mixture were detected on an Infra-red detector (Nicolet 6700, Thermo Fisher Scientific, Waltham, MA) using the KBr disc technique. The measurements were conducted with the scanning wavelength ranging from 4000 to 400 cm^−1^ at ambient temperature.

#### 2.6.2. SEM

The surface morphology of PM, HP-β-CD, PM/HP-β-CD inclusion complex, and PM/HP-β-CD physical mixture was evaluated by SEM. The samples were sprayed with a thin layer of gold and detected by a scanning electron microscope (JEOL, JSM-7500F) operated at 5.0 kV.

#### 2.6.3. ^1^H-NMR

PM, HP-β-CD, and the inclusion were dissolved in 0.6 mL of DMSO-d6 and then analysed by Varian 400 spectrometer (Varian, USA).

### 2.7. *In vitro* anti-tumour studies

#### 2.7.1. *In vitro* cytotoxicity study

To investigate the anti-tumour proliferation effect of PM/HP-β-CD, *in vitro* cytotoxicity test was performed. After being seeded with 96-well plates, the *in vitro* cultured cells were allowed to acclimate for about 1 h. The experimental group was added with 100 μL of medium containing free PM, HP-β-CD, or PM/HP-β-CD with different concentrations, and the control group was added with an equal volume of fresh medium. Each group was set up with 3 parallel holes and a cultured incubator with 5% CO_2_ at 37 °C. At 48 h, the survival rate of each group was calculated with the MTT method, and the IC_50_ value was fitted.

#### 2.7.2. *In vitro* antitumour study

The pro-apoptotic effect of the PM/HP-β-CD complex on SU-DHL-6 cells was investigated by flow cytometry. was evaluated using a flow cytometer with Annexin V-FITC/PI staining following the manufacturer’s instructions cells in good condition were collected and inoculated in the six-well plate with 2⋄10^5^ cells per well. After being cultured for 24 h, cells were treated with PM and PM/HP-β-CD inclusion at 3 nM and 6 nM, using LBH589 at 6 nM as the positive control. The cells without drug treatment were the negative control. After 48 h incubation, the cells were then processed by annexin V-FITC/PI staining as the manufacturer’s instructions (KeyGen, 100 assays).

### 2.8. *In vivo* antitumour studies

#### 2.8.1. *In vivo* antitumour effect in SU-DHL-6 subcutaneous model

To better determine the anti-tumour activity of the PM/HP-β-CD complex, the SU-DHL-6 cell line xenografts were established in immunodeficiency mice. The NOD/SCID mice were subcutaneously inoculated 7 × 10^6^ cells per mouse. When the tumour grew to 100-200 mm^3^, the mice were randomly divided into control group, vehicle group (10% HP-β-CD solution), PM/HP-β-CD complex group (2.5, 5, 10, 20 mg/kg), 4 positive control groups (including HyperCVAD group, RTX group, HyperCVAD + RTX group, LBH589 group), and were treated every other day for 14 days. A mimic of HyperCVAD regimen, standard chemotherapy for B cell lymphoma (22,23), especially aggressive subtypes, for example, double-hit lymphoma, was set as a positive control group for comparison (Table 2). Satellite groups were set up in the 10 mg/kg group, 20 mg/kg group, and the Hyper-CVAD group. Animals in the satellite groups were not sacrificed at the end of the treatment and underwent long-term treatment for further investigation. In the survival study, the animal mortality criterion was that the tumour volume reached 3000 mm^3^ or the individual animal died, and the animal survival time was statistically analysed.

**Table 2.** The doses and schedules of the agents of the Hyper-CVAD + RTX group.

Drug	Dose(mg/kg)	Route	Schedule
Cyclophosphamide	10	i.p.	Administrated once on the first day of the 21-day cycles
Vincristine Sulphate	0.125	i.v.	Administrated once on the first day of the 21-day cycles
Doxorubicin Hydrochloride	0.825	i.v.	Administrated once on the first day of the 21-day cycles
Dexamethasone	1	i.p.	Administrated at day 1, day3, day5, day8 and day 12 of the 21-day cycles
Methotrexate	10	i.p.	Administrated at day2, day5, day9 and day12 of the 21-day cycles
Cytarabine	75	i.p.	Administrated at day3, day6, day10 and day13 of the 21-day cycles
Rituximab（RTX）	10	i.p.	BIW (twice per week)

When the experimental period was over, the tumour was weighed and photographed, the main organs and tumours were collected, fixed, and embedded in paraffin for Histological and immunohistochemical analysis.

#### 2.8.2. Histological analysis (H&E)

The fixed normal organs and tumour tissue were sectioned and stained with haematoxylin and eosin. After dehydration, transparency, and sealing, photos were taken by Nikon E800 microscope.

#### 2.8.3. Immunohistochemical detection of AcH4, γH2AX, and Ki-67

The proliferation of tumour cells was detected by immune histochemical staining with the primary antibody of Ki-67 (Lab Vision, MA, USA). DNA double-stranded breaks were investigated by staining with the primary antibody of H2A histone family member X (γH2AX, Upstate Biotechnology, Lake Placid, NY, USA). Primary antibodies of acetyl histone H4 (Ac-H4, Upstate Biotechnology, Lake Placid, NY, USA) were used to detect the acetylation of Histone H4. FITC-labelled secondary antibodies (F-0382; Sigma) were applied with a concentration of 1:500. The images were taken with the Nikon E800 scope.

#### 2.8.4. *In vivo* pharmacokinetics studies

Adult SD rats (180–220 g) were used to investigate the *in vivo* kinetics of PM after oral or intravenous administration. The rats were fasted overnight and divided into oral and intravenous groups, containing three male and three female rats in each group. Then, 10 mg/kg of PM was administered in each rat. 0.2 mL of jugular vein blood samples were collected at a predetermined time interval of 0, 5, 15, 30, 60, 120, 240, 360, 480, and 600 min, centrifuged at 1500 rpm for 15 min at 4 °C to separate the plasma, and stored at −80 °C for the analysis by UPLC-MS. After the test, Phoenix WinNonlin software and non-compartmental analysis were used to calculate parameters such as maximum plasma concentration (*C*_max_), plasma clearance (CL), time to achieve maximum plasma concentration (*T*_max_), steady-state apparent distribution volume (*V*_dss_), elimination half-life (t_1/2_), area under the concentration-time curve (AUC), and related bioavailability.

### 2.11. Data analysis

The data were expressed as mean ± SD, and a *p* < .05 was considered statistically significant.

## 3. Results and discussion

### 3.1. Phase solubility analysis

HPLC analysis showed good liner relationship in the concentration ranging from 0.31 to 10.01 μg/mL (*y* = 45.706 x − 7.369, R^2^ = 0.9995). The results show excellent precision, repeatability (RSD = 0.45%), and good recoveries (recovery value between 95.98% and 102.25%). PM displays a characteristic single peak at 5.1 min retention time different from which of HP-β-CD.

**Figure 1.**
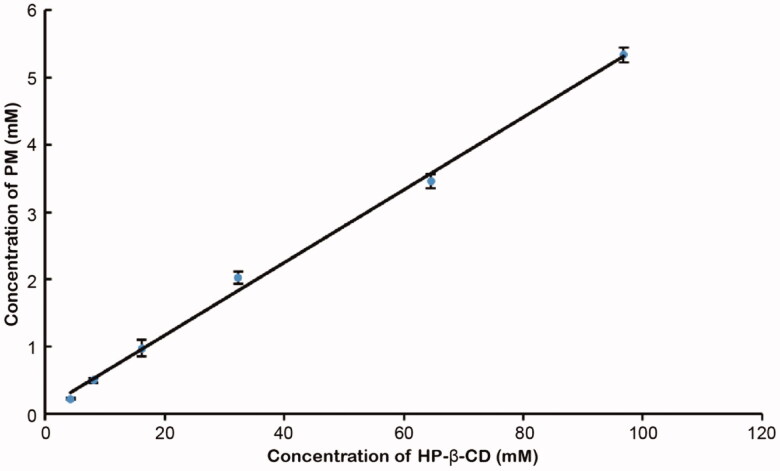
Phase solubility diagram of PM/HP-β-CD system.

The diagram of phase solubility contains not only the molar ratio information but also the Ks of the inclusion complex, which is a key parameter for evaluating the solubility of the inclusion complex [14,16]. It can be observed from Figure 1 that the aqueous solubility of PM had a linear increase with the concentration of HP-β-CD. The solubility curve has a slope of 0.0541 with a correlation coefficient squared value of 0.9970 (*r*^2^>0.990) and can be classified as type AL, which indicates that the solubility increases due to the formation of inclusion at a 1:1 ratio [20]. The detected intrinsic solubility of PM (S0) in pure water at the same temperature is 9.18 μg/mL. The apparent 1:1 Kc is calculated by substituting the slope of the linear phase solubility curve into Equation (1) and is 3642.36 *M*^−1^. The high Kc demonstrates that the PM/HP-β-CD inclusion complex has good stability. Besides, the Gibbs free energy calculated by Equation (2) is negative (−21.15 KJ*mol^−1^), indicating that the process of PM encompassed by cyclodextrin may be spontaneous.

### 3.2. Molecular modelling study

**Figure 2.**
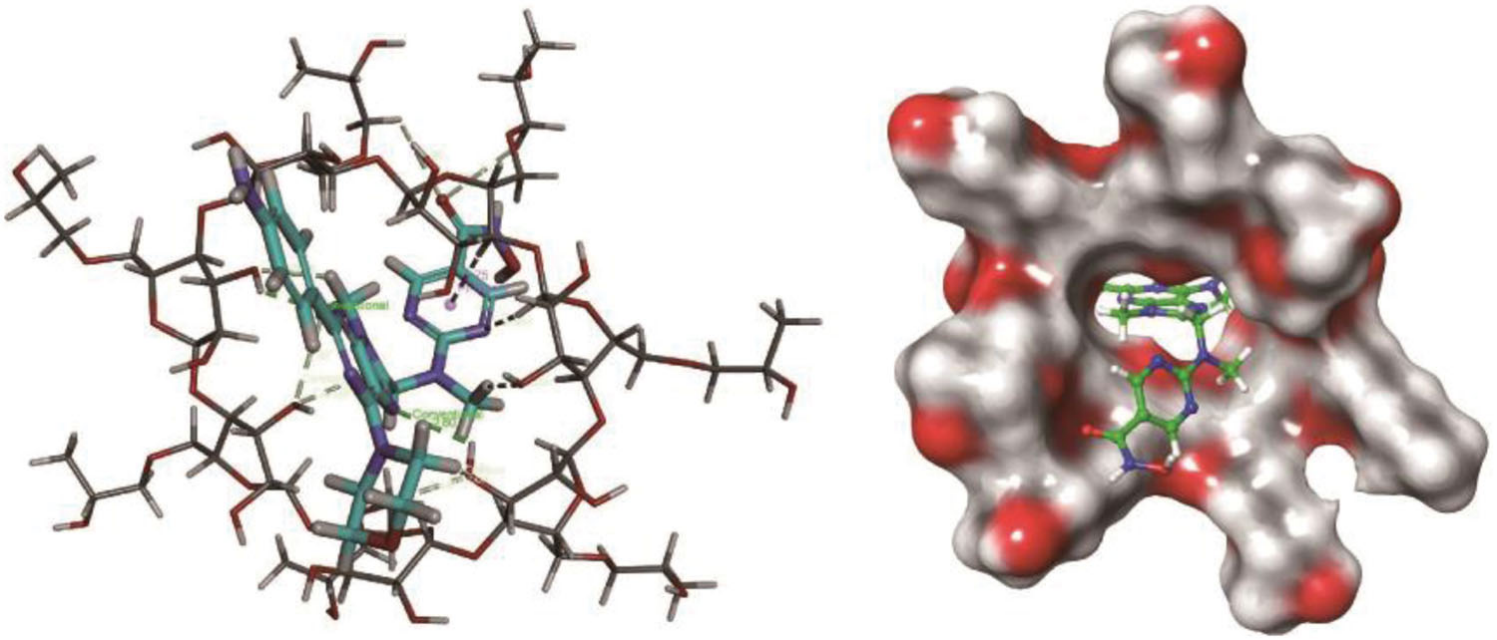
Computer molecular simulation of PM and HP-β-CD.

Under the normal saline environment, the HP-β-CD molecule has a cone-shaped three-dimensional ring structure with a hollow cylinder, and the size and shape of the drug molecule are exactly matched with the inner cavity of the HP-β-CD (Figure 2). The drug molecule binds to the centre of the ring to form a relatively stable complex. The nitrogen atom of the drug molecule forms a plurality of hydrogen bonds with the plurality of hydroxyl groups on the inner side of the HP-β-CD molecule, and the hydrogen atom on the partial ring forms a hydrophobic interaction with the aromatic ring structure of the drug molecule to form Pi-sigma.

Molecular dynamics simulations show that within 20 ns, each bond of the drug molecule has a large torsional motion, but there is only a relatively small fluctuation in the RMSD, the total complex energy, and potential energy, indicating the high stability of the PM/HP-β-CD complex (Figure S1).

### 3.3. Characterization of PM/HP-β-CD complex

**Figure 3.**
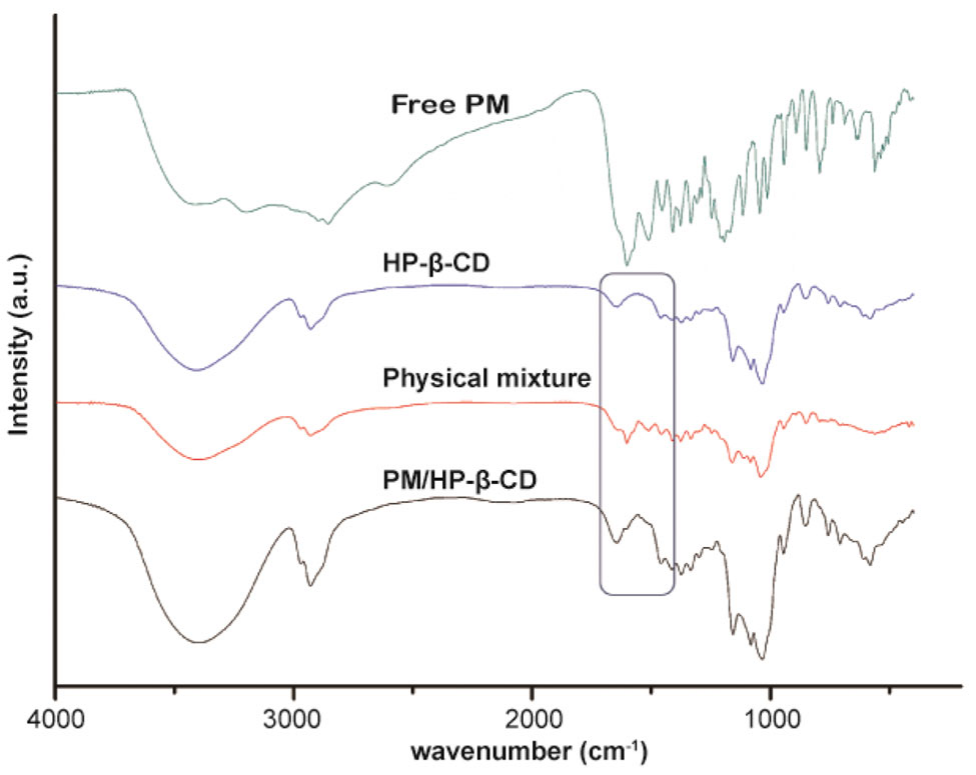
FT-IR spectra of PM, HP-β-CD, PM/HP-β-CD and the physical mixture (1:1 molar ratio).

The FT-IR spectra for PM, HP-β-CD, PM/HP-β-CD inclusion, and physical mixture are displayed in Figure 3. The key frequencies for HP-β-CD are recorded at 3406.29, 2929.10, 1157.68, and 1103.92 cm^−1^, representing the stretching vibration of -OH, the vibration of CH_2_, the C-C vibration, and the O-H bending vibration respectively. For PM, the intense absorption band appearing at 1601.29 cm^−1^ is related to the C=O stretching vibration of the hydroxylamine, which is attributed to the characteristic absorption of hydroxamic acid. The benzene ring C=C stretching vibration found at 1510.28 cm^−1^ is attributed to 1, 4-disubstituted benzene ring. The sharp absorption at 1116.62 and 1045.28 cm^−1^ is the saturated bending vibration, corresponding to the characteristic absorption of morpholine ring.

For the inclusion, due to the low molar ratio of PM/HP-β-CD (1：20), the infra-red absorption spectrum is very similar to that of HP-β-CD. However, we can still find C=O stretching vibration absorption at 1602 cm^−1^, indicating PM is in PM/HP-β-CD. Besides, no extra peaks are found in PM/HP-β-CD, suggesting no chemical reaction occurs. In the physical mixture, multiple absorptions belonging to PM are easy to be found, indicating that PM exists in a free state without any reaction with HP-β-CD.

**Figure 4.**
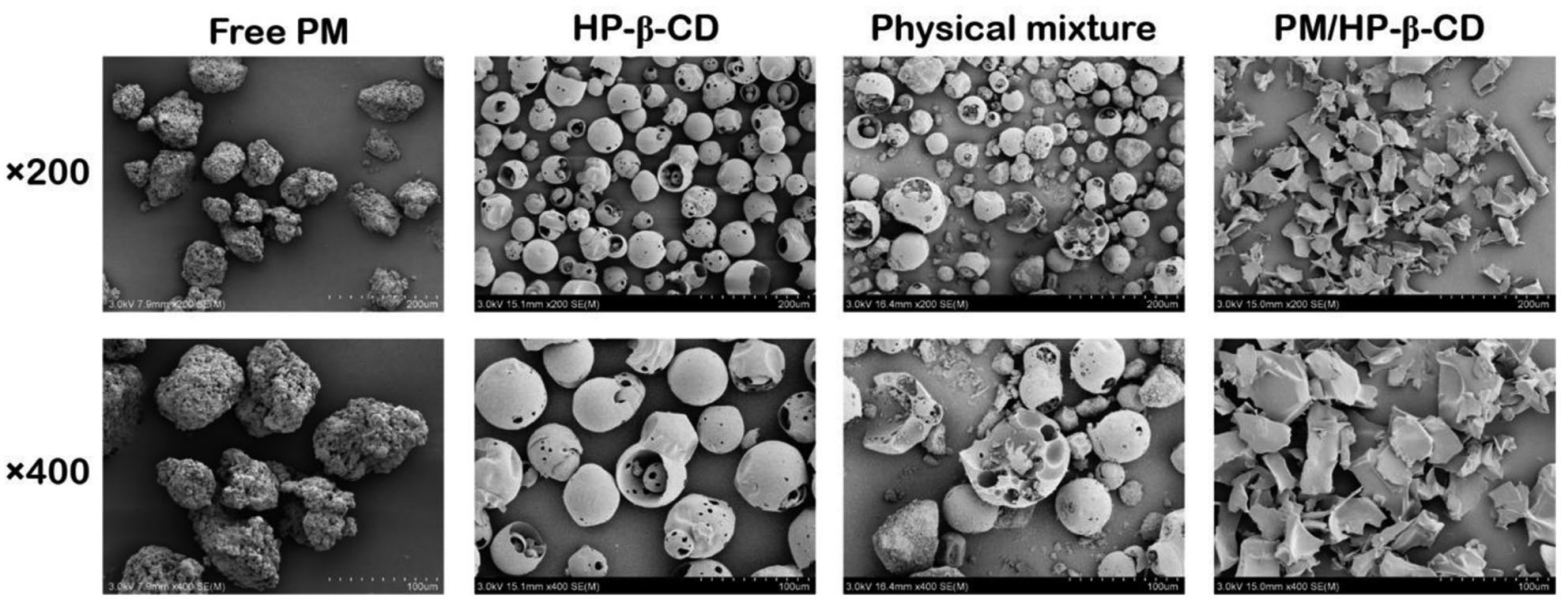
Scanning electron microphotographs of PM, HP-β-CD, the physical mixture (1:1 molar ratio), and PM/HP-β-CD.

Since the surface area enhancement can lead to solubility increment, we observed surface area by SEM. As Figure 4 shows, the free PM appears as amorphous particles, while HP-β-CD displays hollow spherical particles. When the amorphous powder PM is physically mixed with hollow spherical HP-β-CD, PM in the hollow sphere can be observed, indicating that the PM particle size is small enough to be embedded in the HP-β-CD cavity, which is the same with the computer simulation results. Smooth bulk is observed in the complex samples with a large surface area, indicating that the PM could be embedded in the HP-β-CD to improve water solubility. Thus, the morphology of PM and HP-β-CD changes significantly during inclusion formation.

**Figure 5.**
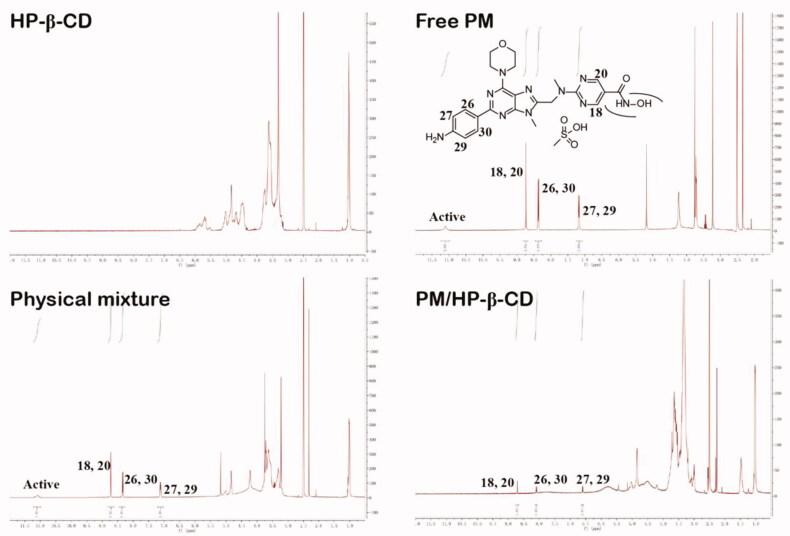
1H-NMR spectra and chemical structural formula of PM, HP-β-CD, PM/HP-β-CD and the physical mixture (1:1 molar ratio).

The ^1^H-NMR was also used to confirm the interactions between PM and HP-β-CD (Figure 5). The spectra of the latter correspond to the research published by Ma et al. [24]. The spectrum of PM showed that hydrogen at the position of 8.73 ppm (d, 2H) is assigned to positions 18, 20 of the pyrimidine ring and has no significant shift in the physical mixture or PM/HP-β-CD. However, the hydrogen signals at the 26&30 (8.36 ppm, d, 2H), 27&29 (7.18 ppm, s, 2H) position of the aminobenzene ring undergo a significant blue shift in PM/HP-β-CD, a phenomenon that does not occur in the physical mixture. These results showed that the side of the aminobenzene ring in the PM is deeply embedded into the HP-β-CD cavity, while the side of the pyridine ring is slightly embedded or unsuccessfully embedded. Thus, PM/HP-β-CD enhances the solubility of PM in water. Chemical shift change values are shown in Table 3.

**Table 3.** Chemical shift (*δ*, ppm*)* change values relating to the signals of PM in different states.

Site	PM	Physical mixture	PM/HP-β-CD	*Δ δ*
18, 20	8.73	8.72	8.71	−0.02
26, 30	8.36	8.34	8.10	−0.26
27, 29	7.17	7.12	6.60	−0.57

### 3.4. *In vitro* antitumour efficacy

**Figure 6.**
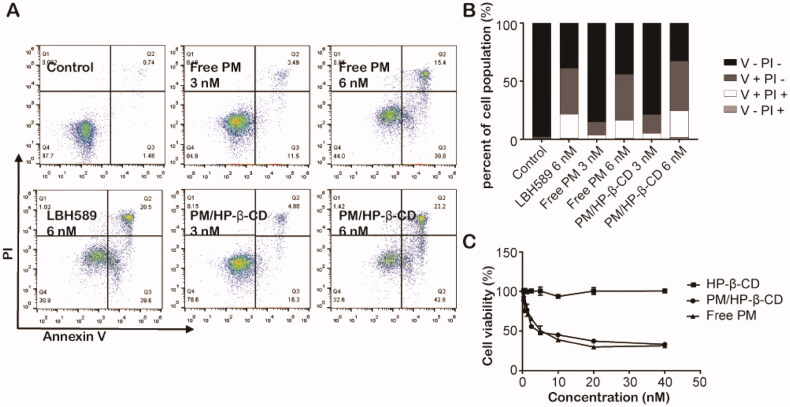
*In vitro* antitumour effect. (A) Free PM and PM/HP-β-CD induced apoptosis in SU-DHL-6 cells. (B) A histogram of apoptosis data. (C) Cell viability of free PM and PM/HP-β-CD on SU-DHL-6 cells.

To investigate apoptosis induced by PM in SU-DHL-6 cells, flow cytometry analysis was performed. As shown in Figure 6(A) and (B), after incubation with 6 nM LBH589, 3 nM, 6 nM PM and PM/HP-β-CD for 48 h, the apoptotic SU-DHL-6 cells is 60.1%, 14.99%, 55.2%, 21.18%, 66%, respectively. These results indicated that PM/HP-β-CD showed a little better efficacy in inducing apoptosis of SU-DHL-6 cells than PM.

When the concentration of HP-β-CD increases, cell viability does not change significantly, as shown in Figure 6(C), and even when the given concentration of blank HP-β-CD solution reaches 799.6 nM, the cell survival rate is still as high as 100.85%. This result demonstrates that the blank HP-β-CD solution does not damage cells. PM and PM/HP-β-CD have similar anti-proliferative curves and the caculatedIC50 of which is 1.88 nM and 2.43 nM, which means that both PM and PM/HP-β-CD inclusion complex has a dose-dependent inhibitory effect on the growth of SU-DHL-6 cells.

### 3.5. *In vivo* antitumour efficacy

**Figure 7.**
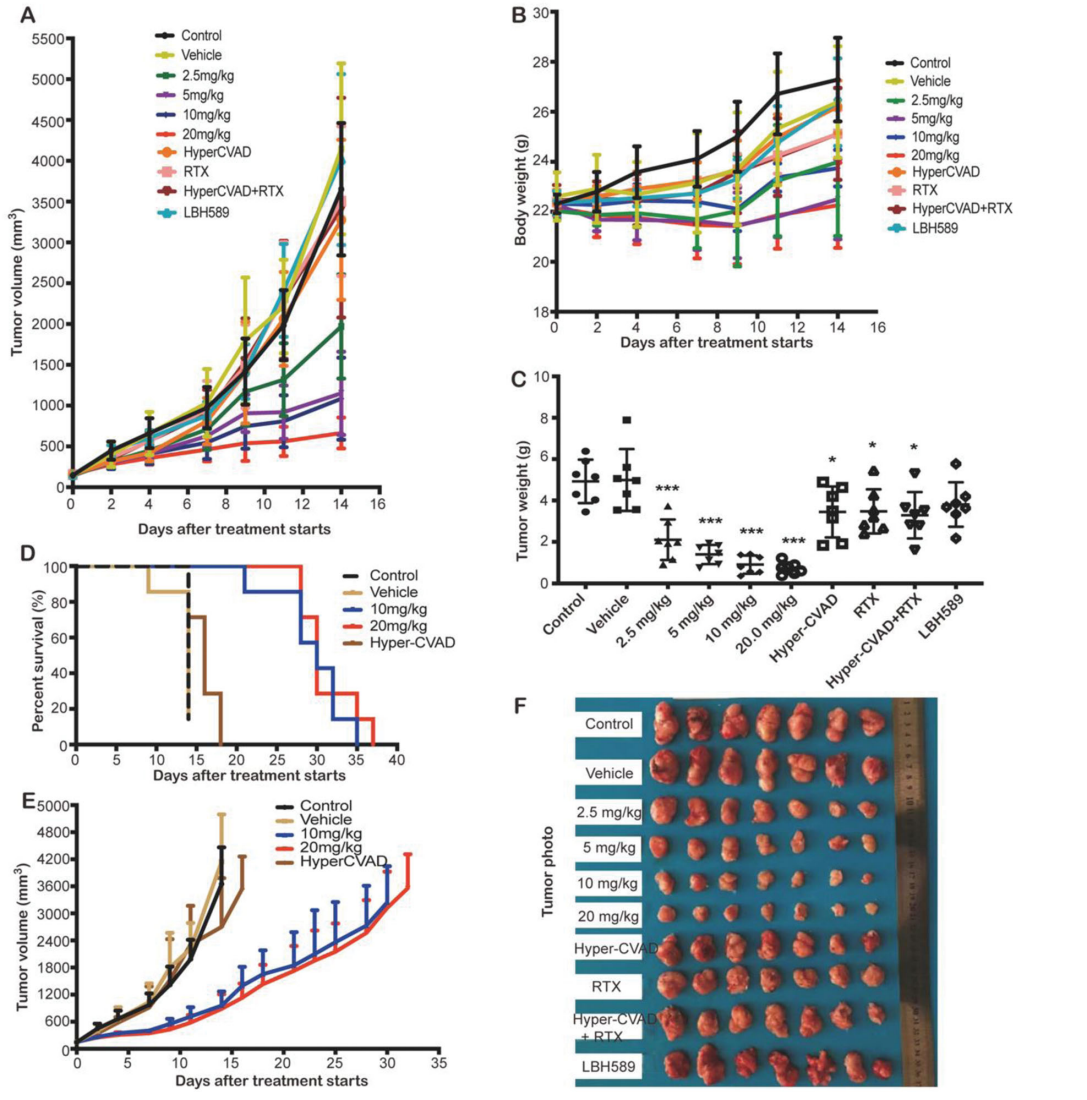
*In vivo* anti-tumour effect in SU-DHL-6 xenograft subcutaneous tumour model. (A) Growth curve of the tumour. (B) Bodyweight of the mice in each group. (C) Tumour weight of each group. (D) Survival curve of each group during survival investigation. (E) Tumour growth curves in each group during survival investigation. (F) Photographs of subcutaneous tumours in each group.

In an immunodeficiency mouse model of SU-DHL-6 cell line xenograft, PM/HP-β-CD was intravenously administered with doses of 2.5 mg/kg, 5 mg/kg, 10 mg/kg, and 20 mg/kg three times a week. The results are displayed in Figure 7 and Table 4, where the tumour inhibition rate is significantly increased with the dose increase. The tumour inhibition rate of PM/HP-β-CD at 2.5 mg/kg is 57.35% while that of Hyper-CVAD/RTX positive control is only 33.28%. The results show that PM/HP-β-CD can dose-independently inhibit the growth superior to current clinical protocols in SU-DHL-6 subcutaneous models, and a low dose of PM/HP-β-CD showed better anti-tumour efficacy than Hyper-CVAD/RTX.

**Table 4.** Antitumour effects of PM/HP-β-CD in human double-hit lymphoma SU-DHL-6 xenograft tumours.

Group	Dose mg/kg	Number	Body weight (g) X ± SD		Tumour volume (mm^3^)			RTV	Tumour weight (g)	
		End/ begin			X ± SD		T/C%			
			Begin	End	Begin	End			X ± SD	Inhibition (%)
Control	–	7/7	22.30 ± 0.4	27.3 ± 1.7	144.9 ± 19.1	3651.3 ± 811.7	–	26.06	4.92 ± 1.05	–
Vehicle	–	7/7	22.60 ± 1.0	26.4 ± 2.2	148.1 ± 19.1	4146.1 ± 1047.3	108.63	28.31	4.99 ± 1.49	−1.33
LBH589	–	7/7	22.4 ± 0.5	26.4 ± 1.8	152.7 ± 30.6	4015.5 ± 1048.0	101.83	26.54	3.81 ± 1.08	22.72
HyperCVAD	–	7/7	22.4 ± 0.5	26.2 ± 1.1	154.7 ± 36.9	3276.8 ± 981.2	82.03	21.38	3.44 ± 1.23^a^	30.08
RTX	10	7/7	22.4 ± 0.4	25.1 ± 1.0	153.7 ± 33.1	3505.3 ± 917.1	92.71	24.16	3.47 ± 1.07^ad^	29.47
HyperCVAD + RTX	–	7/7	22.5 ± 0.6	25.1 ± 1.8	155.2 ± 38.1	3425.4 ± 1347.2	83.29	21.71	3.29 ± 1.12^ad^	33.28
PM	2.5	7/7	22.0 ± 0.3	24.0 ± 3.0	140.3 ± 23.7	1970.1 ± 638.3	54.53	14.21^begkmp^	2.10 ± 0.98^cekmp^	57.35
	5	7/7	22.3 ± 0.5	22.5 ± 1.6	146.2 ± 26.5	1150.0 ± 510.6	31.69	8.26^cfiloq^	1.39 ± 0.47^cfhlnr^	71.80
	10	7/7	22.3 ± 0.5	23.7 ± 0.7	150.9 ± 32.6	1084.2 ± 502.8	28.37	7.39 ^cfiloq^	0.90 ± 0.43^cfilor^	81.67
	20	7/7	22.2 ± 0.3	22.3 ± 1.7	158.9 ± 22.6	664.3 ± 190.7	16.53	4.31^cfilor^	0.73 ± 0.28 ^cfilor^	85.20

vs Control, ^a^*p* < .05; ^b^*p* < .01; ^c^ *p* < .001；vs Vehicle, ^d^*p* < .05; ^e^*p* < .01; ^f^ *p* < .001；vs HyperCVAD + RTX, ^g^*p* < .05; ^h^*p* < .01; ^i^*p* < .001; vs LBH589, ^j^*p* < .05; ^k^*p* < .01; ^l^*p* < .001; vs Hyper-CVAD, ^m^*p* < .05; ^n^*p* < .01; ^o^*p* < .001; vs RTX ^p^*p* < .05; ^q^*p* < .01; ^r^*p* < .001.

H&E staining analysis of the main internal organs shows that no serious damage occurs after 14 days of treatment with PM/HP-β-CD (Figure S2A). To explore the possible mechanism of PM exerting the drug effect *in vivo*, the expression of Ac-H_4_, γH_2_AX, and Ki-67 in the tumour tissue was detected by immunostaining [25]. Results (Figure S2B) showed that expression of Ac-H_4_ and γH_2_AX increased, and which of Ki-67 decreased after PM treatment. These results showed that PM may exert its tumour-inhibiting effect by increasing histone H4 acetylation, DNA double-stranded breaks, and inhibiting proliferation of tumour cells. Importantly, our results also showed that the effect of PM/HP-β-CD (20 mg/kg) was superior to that of the Hyper-CVAD/RTX combined administration (Figure S2B).

In the survival study, the median survival time of the control group, the vehicle group, the Hyper-CVAD group, and the PM/HP-β-CD group (10, 20 mg/kg) is 14, 14, 16, and 30 days, respectively (Table 5), indicating that PM/HP-β-CD has long-term inhibitory activity and better antitumor effect on subcutaneous tumours.

**Table 5.** Effect of PM for Injection on median survival of subcutaneously transplanted mice with human Burkitt's lymphoma Raji cell line.

Group	Control	Vehicle	PM 10mg/kg	PM 20mg/kg	HyperCVAD
Median survival (day)	14	14	30^bei^	30 ^bei^	16

vs Control, ^a^*p* < .05; ^b^*p* < .01; ^c^ *p* < .001; vs Vehicle, ^d^*p* < .05; ^e^*p* < .01; ^f^*p* < .001; vs Hyper-CVAD, ^g^*p* < .05; ^h^*p* < .01; ^i^*p* < .001.

### 3.6. *In vivo* pharmacokinetic study

The plasma concentration–time curves of PM after oral and intravenous administration are shown in Figure S3, and the relevant pharmacokinetic parameters are shown in Table 6. These results showed that the plasma concentration of PM in the intravenous administration group is much higher than which in the oral administration group in the previous hours, and the maximum value (C_max_) is 3137.0 ± 1323.0 ng/mL and 49.3 ± 26.4 ng/mL, respectively. The AUC in the intravenous administration group was nearly 16 times higher than that in the oral administration group (837.0 ± 277.0 vs 56.2 ± 24.6 ng/mL), so the calculated bioavailability of PM was 6.71%. In addition, the i.v. administration rats group showed lower plasma clearance (CL) and a shorter plasma half-time of the drug (t_1/2_) than the oral administration group. These results demonstrate that a large amount of PM was cleared by the organism after oral administration, leading to insufficient efficacy. Our studies show that after preparation of PM/HP-β-CD complex, the pharmacokinetics properties by intravenous administration improved a lot with higher AUC, C_max_, and lower CL than oral administration. Anti-tumour efficacy studies between the two administration routes also showed that i.v. delivery had a better anti-tumour effect *in vivo* (Figure S4).

**Table 6.** Pharmacokinetic parameters of oral administration and intravenous administration PM solution at a dose of 10 mg/kg.

	p.o.	i.v.
C_max_ (μg/L)	49.3 ± 26.443	3137 ± 1323
T_max_ (min)	5.67 ± 1.97	4.67 ± 1.63
AUC (μg/L∗ h)	56.2 ± 24.57	837 ± 277
t_1/2_ (h)	1.62 ± 0.691	0.340 ± 0.328
CL (L/h/Kg)	437 ± 197.44	13.5 ± 6.10

## 4. Conclusion

In this study, an injectable formulation of PM in HP-β-CD (10% w/v) was prepared to improve its water solubility. The solubility of PM increases due to the formation of inclusion at a 1:1 ratio. *In vitro*, PM/HP-β-CD inclusion showed an inhibitory effect on the growth of SU-DHL-6 cells. *In vivo*, intravenous administration of PM showed a much better therapeutic effect compared with LBH589 and Hyper-CVAD/RTX, avoiding poor bioavailability of oral administration. Our research provides a way for clinical administration of PM, which has been under phase I clinical trial for the treatment of relapsed or refractory B-cell-related haematologic malignancies in China and the USA.

## Supplementary Material

Supplemental Material

## Data Availability

The authors confirm that the data supporting the findings of this study are available within the article and its supplementary materials.
