# Supplementary material for: Therapeutic efficacy of an injectable formulation of purinostat mesylate in SU-DHL-6 tumour model
Source: Ann Med. 2022 Mar 4;54(1):743–53. doi: 10.1080/07853890.2022.2045347 (PMC8903780; doi:10.1080/07853890.2022.2045347)
Supplement: Supplemental Material [file IANN_A_2045347_SM0345.zip › Supporting information/PM article support information 20210806.docx]

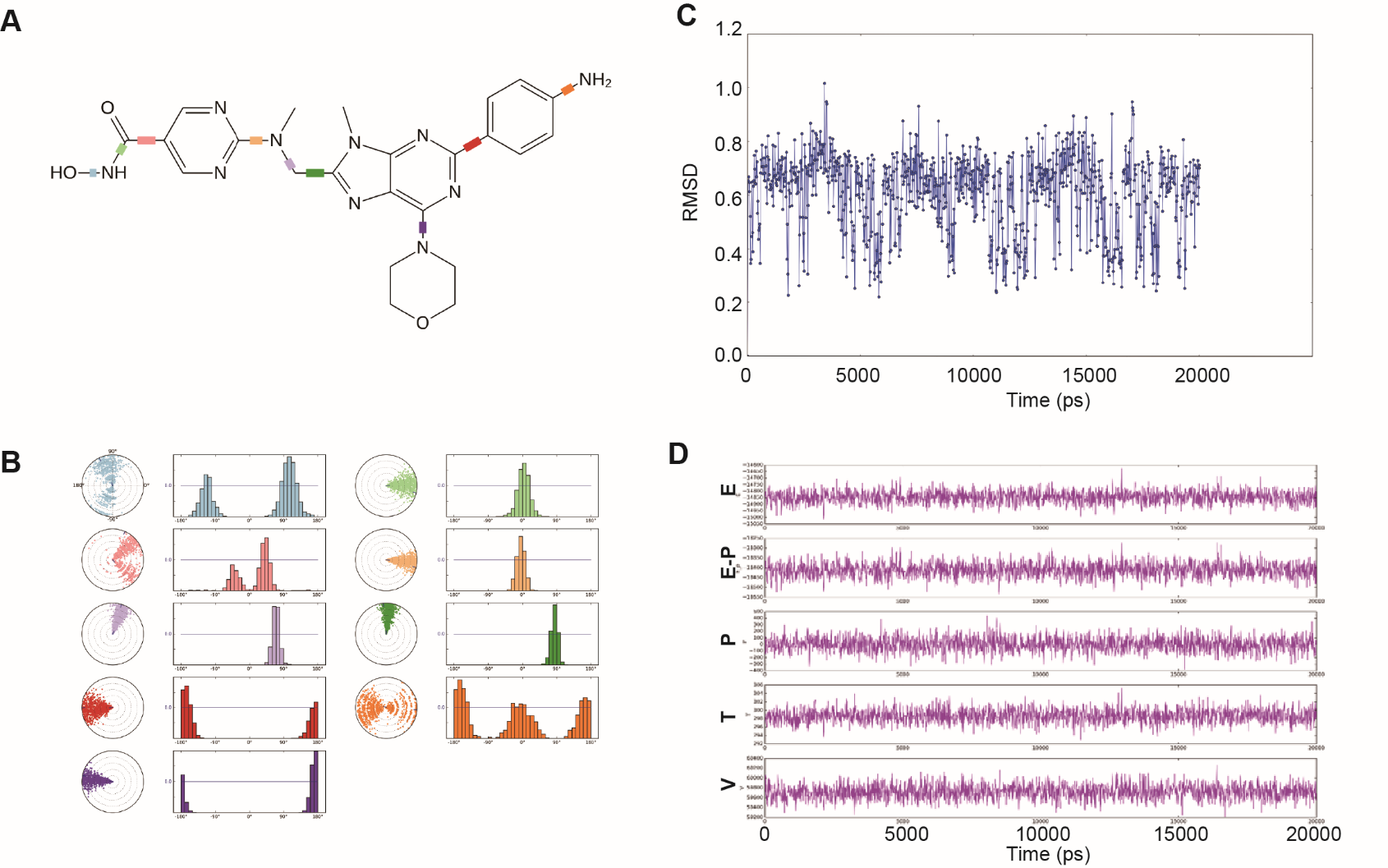


Figure S1 (A) The 2D schematic of a ligand with color-coded rotatable bonds; (B) Each rotatable bond torsion is accompanied by a dial plot and bar plots of the same color; (C) Root mean square deviation of a ligand concerning the reference conformation (typically the first frame is used as the reference and it is regarded as time t=0); (D) System energy parameter. E, E-P, P, T, V stands for total energy, potential energy, pressure, temperature, the velocity of the system.


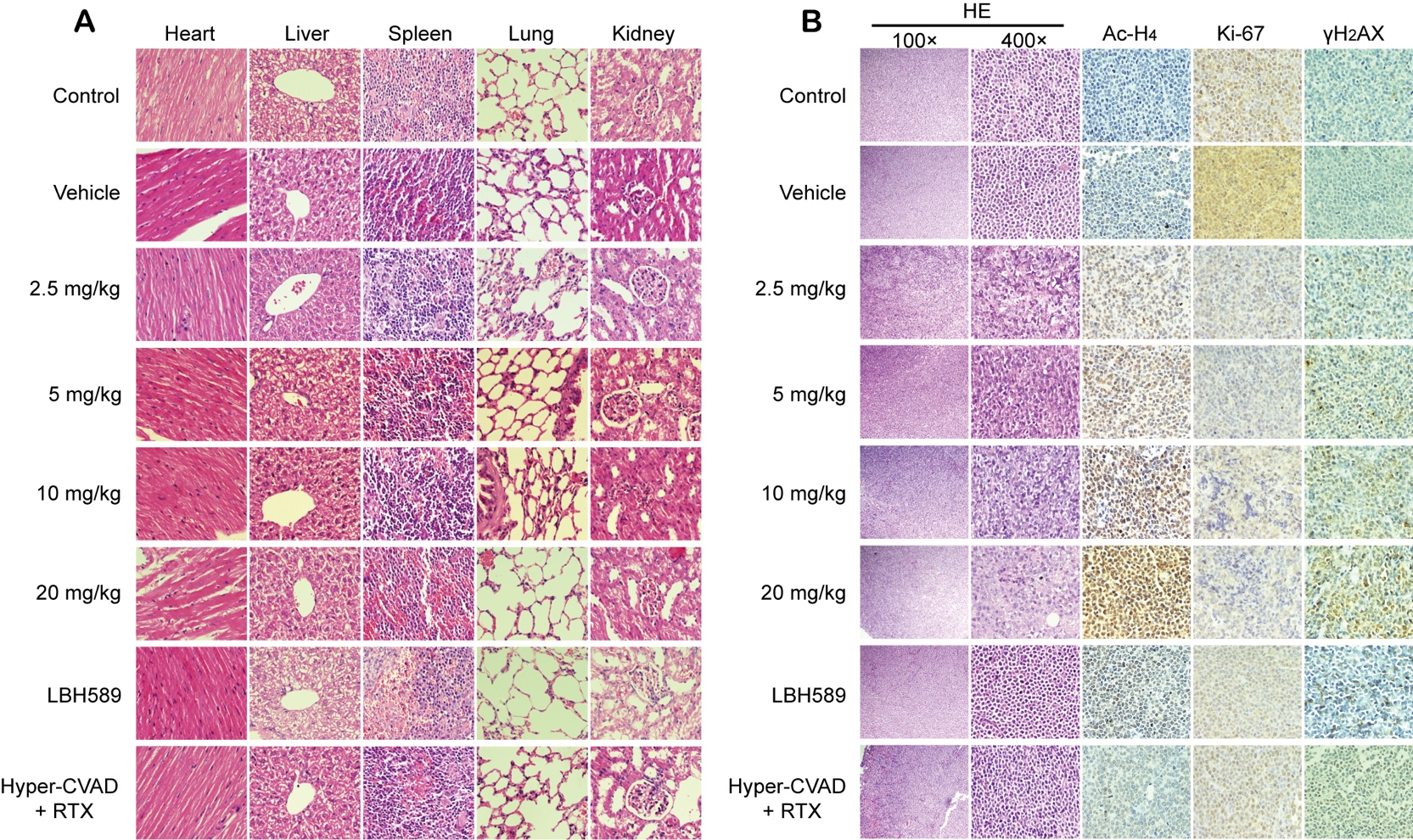


Figure S2 (A) H＆E staining (×400 times) of the main organs of animals with SU-DHL-6 subcutaneous tumor; (B) H＆E staining (×100, 400 times)and immunohistochemical staining (×400 times) of SU-DHL-6 subcutaneous tumor


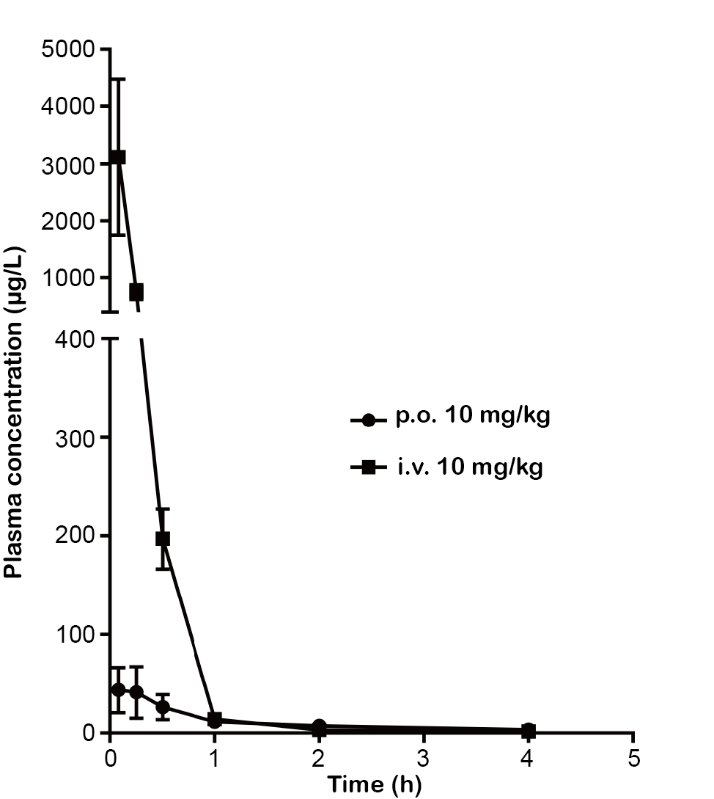


Figure S3 Plasma concentration-time profiles of PM in rats after intravenous injection of PM/HP-β-CD and oral administration of PM suspension


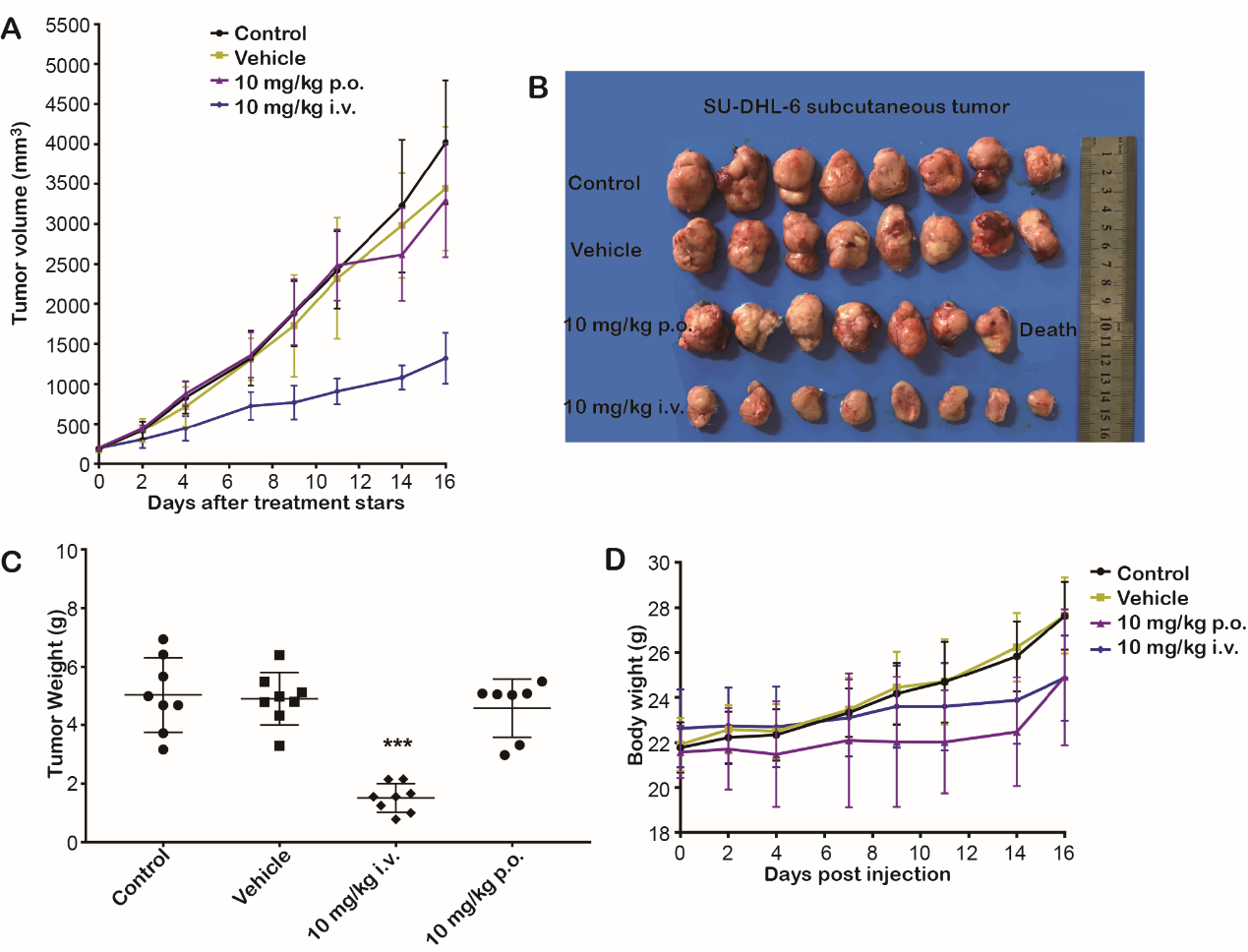


Figure S4 *In vivo* anti-tumor effect in SU-DHL-6 xenograft subcutaneous tumor model. (A) Growth curve of the tumor. (B) Photographs of subcutaneous tumors in each group. (C) Tumor weight of each group. (D) Bodyweight of the mice in each group
