# Supplementary material for: Therapeutic efficacy of an injectable formulation of purinostat mesylate in SU-DHL-6 tumour model
Source: Ann Med. 2022 Mar 4;54(1):743–53. doi: 10.1080/07853890.2022.2045347 (PMC8903780; doi:10.1080/07853890.2022.2045347)
Supplement: Supplemental Material [file IANN_A_2045347_SM0345.zip › Supporting information/PM article support information 20220104.docx]

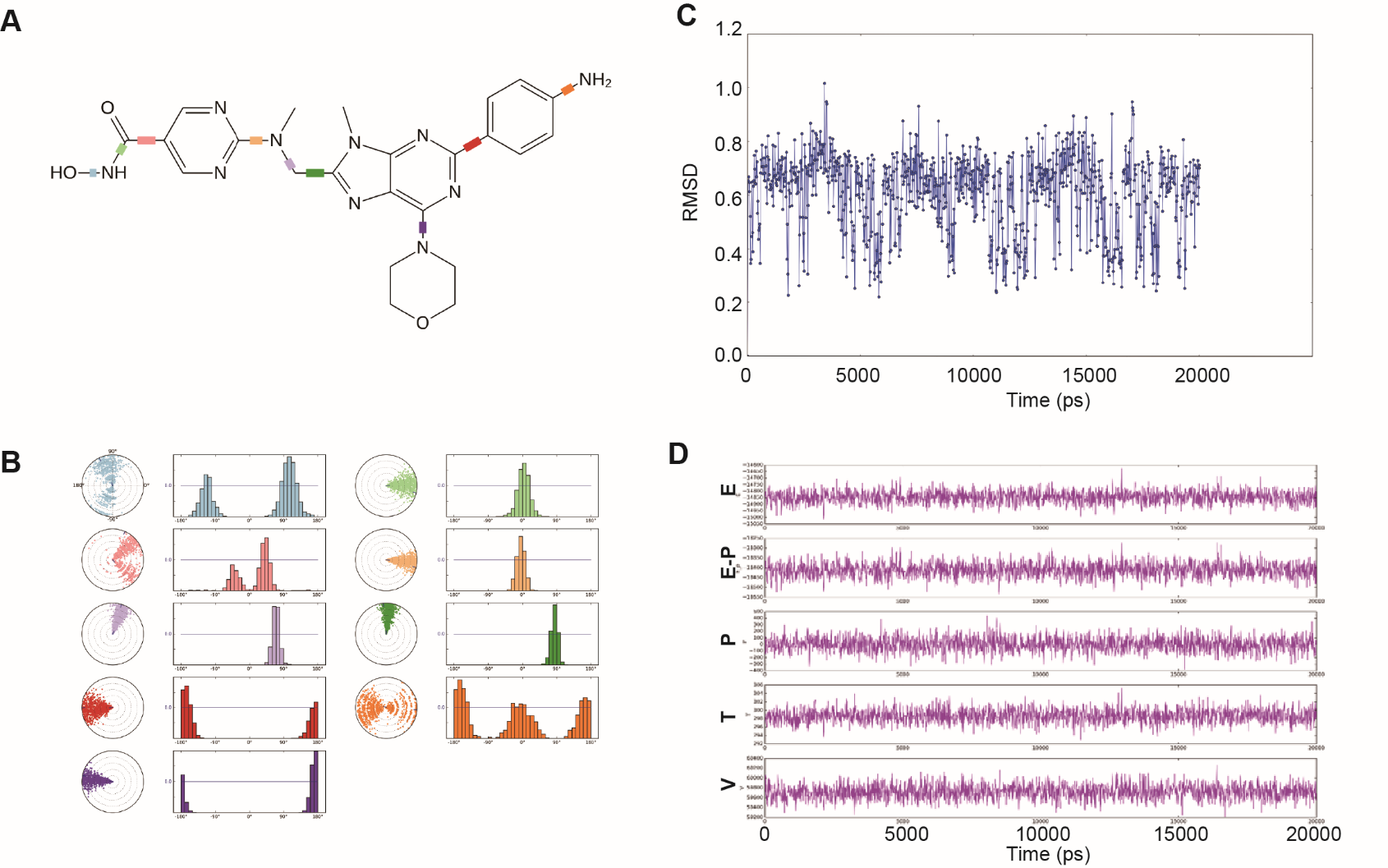


Figure S1 (A) The 2D schematic of a ligand with color-coded rotatable bonds; (B) Each rotatable bond torsion is accompanied by a dial plot and bar plots of the same color; (C) Root mean square deviation of a ligand concerning the reference conformation (typically the first frame is used as the reference and it is regarded as time t=0); (D) System energy parameter. E, E-P, P, T, V stands for total energy, potential energy, pressure, temperature, the velocity of the system.


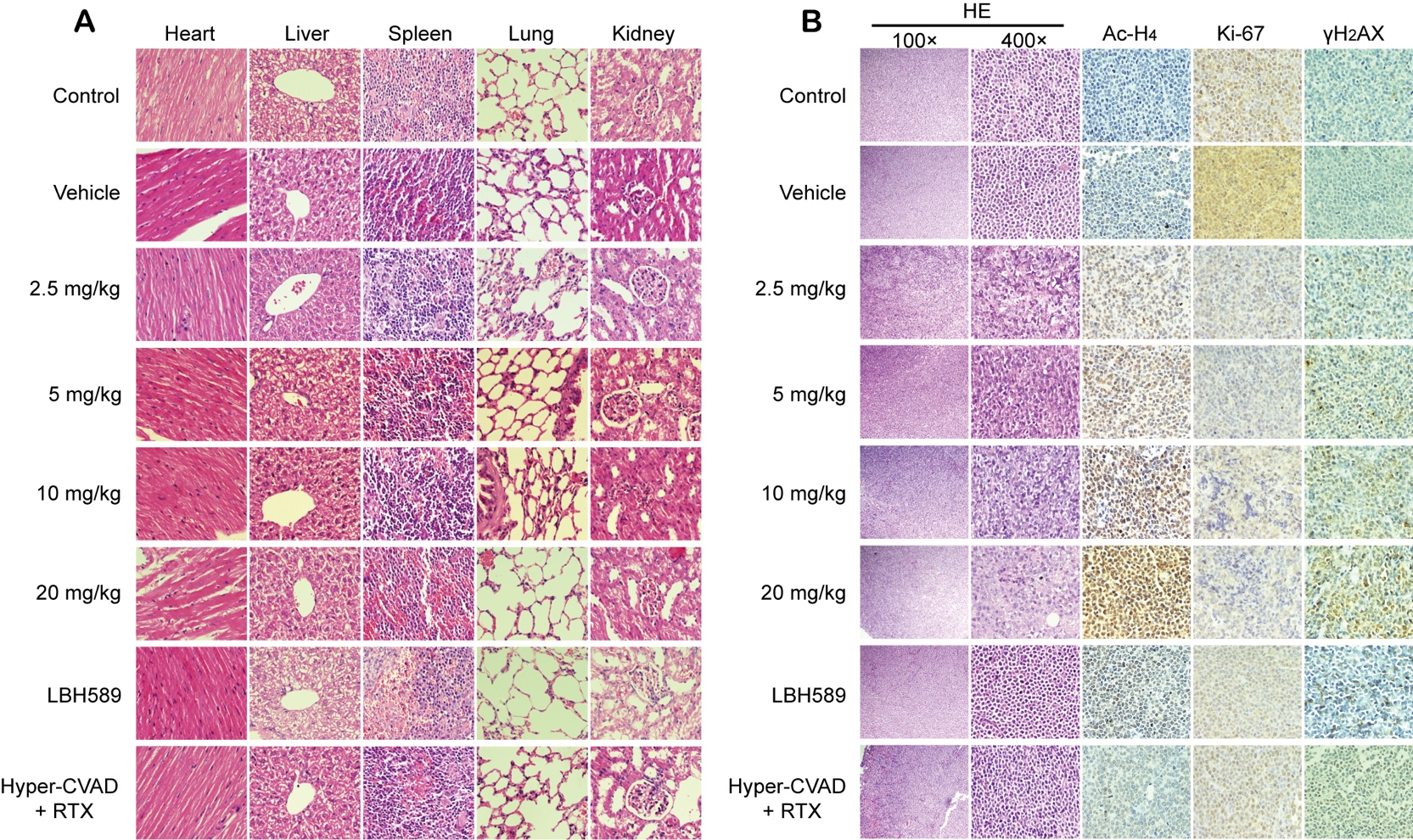


Figure S2 (A) H＆E staining (×400 times) of the main organs of animals with SU-DHL-6 subcutaneous tumor; (B) H＆E staining (×100, 400 times)and immunohistochemical staining (×400 times) of SU-DHL-6 subcutaneous tumor


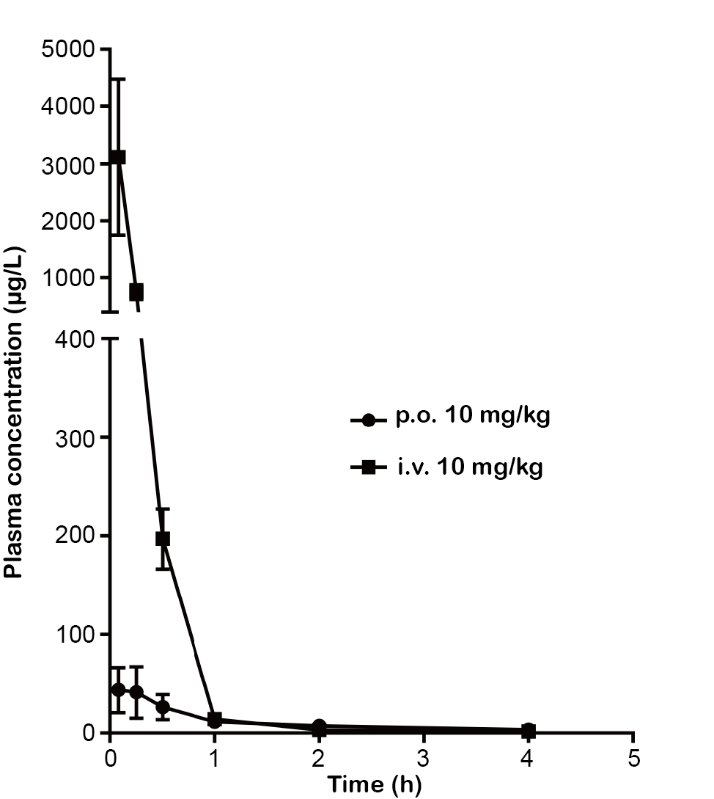


Figure S3 Plasma concentration-time profiles of PM in rats after intravenous injection of PM/HP-β-CD and oral administration of PM suspension

**Oral administration versus intravenous administration study**

To determine whether PM is suitable for oral or intravenous administration, the anti-tumor activity and the safety were investigated in a human double-hit lymphoma SU-DHL-6 cell line xenografts established in immunodeficiency mice. Each mouse was injected subcutaneously with 0.1 mL of cell suspension containing 1×10^6^ cells. When the tumor volume was approximately 200-300 mm^3^, the mice were randomly divided into control group, vehicle group (10% HP-β-CD solution), PM suspension group (10 mg/kg, p.o.), PM/HP-β-CD group (10 mg/kg, i.v.), and were treated every other day for 16 days. Tumor size and body weight were measured during the experimental period and the tumor volumes were calculated according to the formula: volume (mm^3^) = length × width^2^ × 0.5. When the experimental period was over, all the mice were sacrificed, and the tumor was weighed and photographed.

**Table S1 Inhibition of SUDHL-6 Transplanted Tumor by Intravenous and Oral Administration of Purinostat Mesylate**

| **Group** | **Dose**  **mg/kg** | **Number** | **Body Weight (g)**  **X ±SD** | | **Tumor volume (mm^3^)** | | | **RTV** | **Tumor Weight (g)** | |
| --- | --- | --- | --- | --- | --- | --- | --- | --- | --- | --- |
|  |  | **End /Begin** |  |  | **X ±SD** | | **T/C%** |  |  |  |
|  |  |  | **Begin** | **End** | **Begin** | **End** |  |  | **X ±SD** | **Inhibition**  **(%)** |
| Control | - | 8/8 | 21.8±1.1 | 27.6±1.5 | 185.2±26.5 | 4020.6±774.7 | - | 22.15 | 5.03±1.28 | - |
| Vehicle | - | 8/8 | 21.9±1.2 | 27.7±1.7 | 176.3±33.5 | 3442.8±775.1 | 88.22 | 19.54 | 4.89±0.89 | 2.69 |
| i.v. | 10 | 8/8 | 22.6±1.7 | 24.9±1.9 | 194.6±26.1 | 1321.9±317.2 | 30.87 | 6.84^cfi^ | 1.51±0.49^cfi^ | 69.96 |
| p.o. | 10 | 7/8 | 21.6±1.2 | 24.9±3.0 | 199.5±22.5 | 3300.6±715.1 | 75.46 | 16.71^a^ | 4.57±0.99 | 9.16 |

vs Control, ^a^P<0.05; ^b^P<0.01; ^c^P<0.001；vs Vehicle, ^d^P<0.05; ^e^P<0.01; ^f^ P<0.001；vs p.o., ^g^P＜0.05; ^h^P<0.01; ^i^P<0.001.

In this study, the subcutaneous tumor model of the human dual-strength lymphoma SU-DHL-6 cell line was established in the NOD/SCID mice, and the experimental results are shown in Figure S4 and Table S1. When PM was administered intravenously three times a week with a dose of 10 mg/kg, the tumor inhibition rate was 69.96%, and the relative tumor proliferation T/C value was 30.87%. However, the tumor inhibition rate of the oral administration of PM suspension was only 9.16% and the relative tumor proliferation T/C value was 75.46%. Oral administration of PM suspension is significantly less effective than intravenous administration with the same dose and frequency of administration, and the difference is statistically significant. As shown in Figure S4, one of the PM oral groups died, while the venous group did not have animals in abnormal status. Besides, it can be seen that while the tumor volume increase in the PM group was not significantly inhibited, the weight gain of the animals was significantly lower than that of the blank control or the vehicle group, also indicating that oral administration is inferior to the intravenous administration.


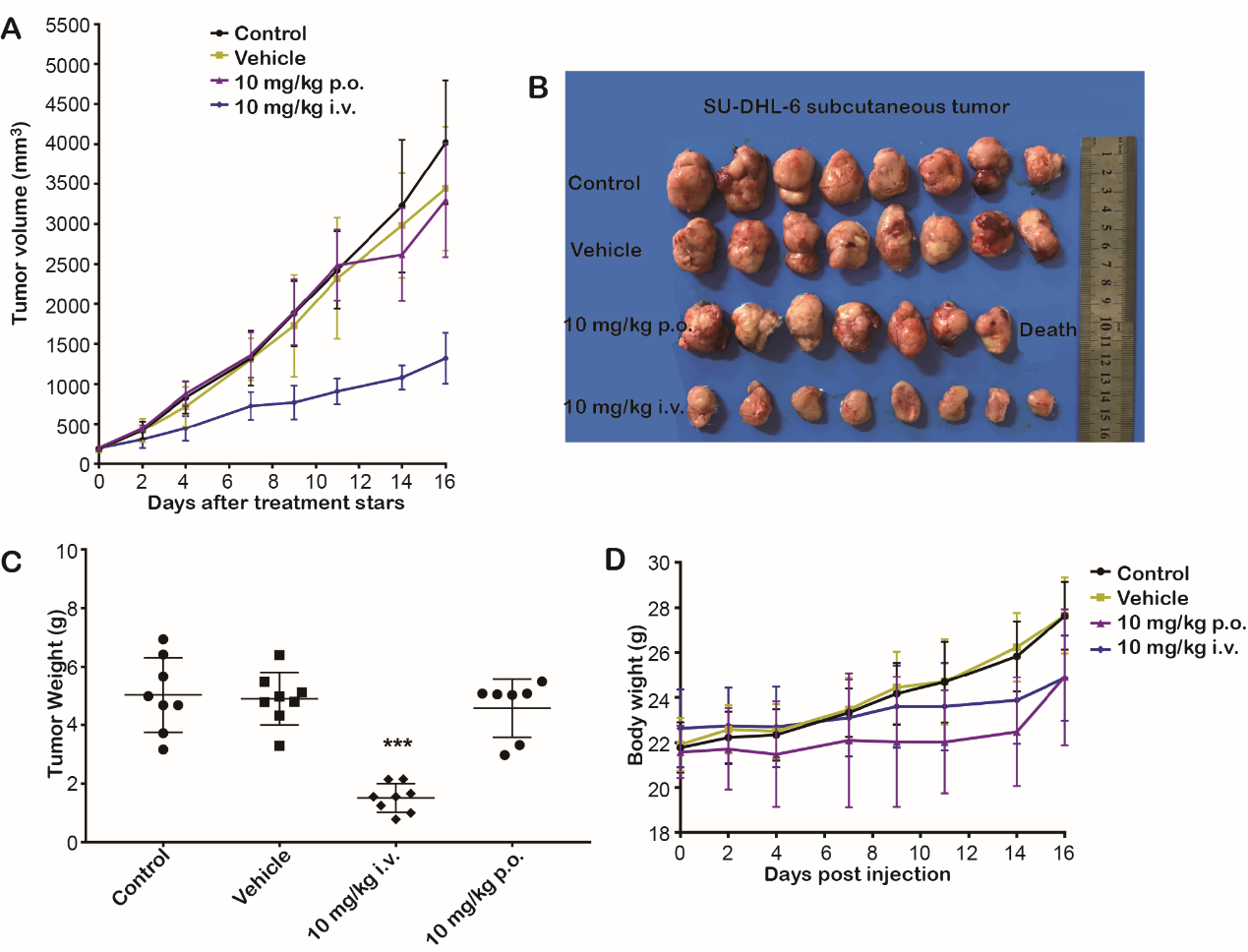


Figure S4 *In vivo* anti-tumor effect in SU-DHL-6 xenograft subcutaneous tumor model. (A) Growth curve of the tumor. (B) Photographs of subcutaneous tumors in each group. (C) Tumor weight of each group. (D) Bodyweight of the mice in each group

**Hemolysis test**

The experiment was carried out in accordance with the 1148 general rule of the fourth part of the Chinese Pharmacopoeia. Five clean glass test tubes were taken, the corresponding solution was added according to Table S2, and immediately placed in an incubator of 37 ± 0.5 ℃ for incubation. Hemolysis and coagulation reaction were observed 3 hours later.

Table S2 Solutions added to different test tubes

| Test tube numbers | 1、2 | 3 | 4 | 5 |
| --- | --- | --- | --- | --- |
| 2% red blood cell suspension (mL) | 2.5 | 2.5 | 2.5 |  |
| 0.9% sodium chloride solution (mL) | 2.2 | 2.5 |  | 4.7 |
| Purified water (mL) |  |  | 2.5 |  |
| PM/HP-β-CD solution (mL) | 0.3 |  |  | 0.3 |

As shown in Figure S5, no obvious hemolysis occurred in test tubes 1 and 2 compared with that in test tube 3, while tube 4 was a positive control with 100% hemolysis.


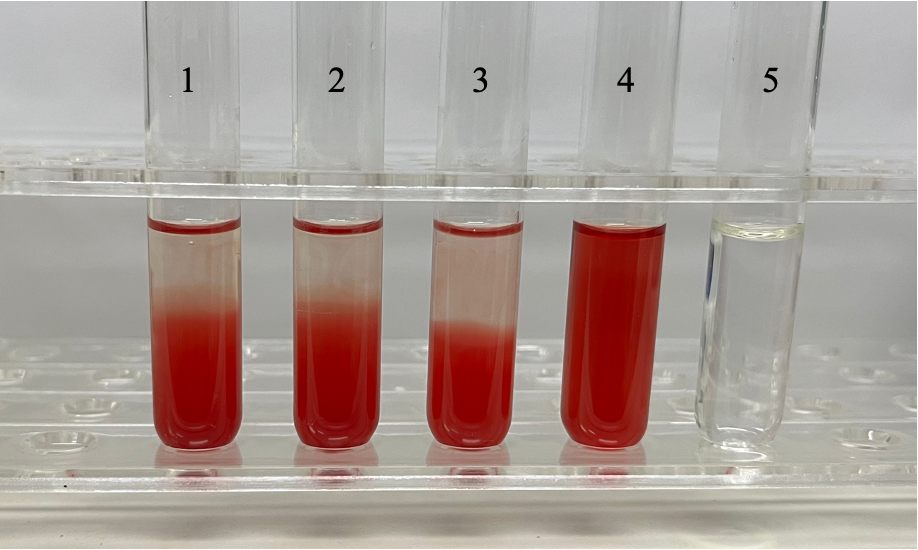


Figure S5 Results of hemolysis test after incubation at 37± 0.5 ℃ for 3 hours. Tube 1 and tube 2 stands for PM/HP-β-CD test samples, tube 3, 4 and 5 respectively stands for negative control, positive control and blank control.
